# An In Vitro Study: Does Adding Iodine Potassium Iodide and Cetrimide to Calcium Hydroxide Paste Enhance Its Antimicrobial Effect Against Oral Biofilms?

**DOI:** 10.7759/cureus.51203

**Published:** 2023-12-27

**Authors:** Hadi M Alamri, He Liu, Duo Zhang, Ya Shen, Markus Haapasalo

**Affiliations:** 1 Department of Dentistry, King Faisal Specialist Hospital and Research Center, Riyadh, SAU; 2 Division of Endodontics, Department of Oral Biological and Medical Sciences, University of British Columbia, Vancouver, CAN

**Keywords:** iodine potassium iodide, cetrimide, calcium hydroxide, biofilm, antibacterial agents

## Abstract

Objectives

This study aimed to evaluate the antibiofilm effect of calcium hydroxide (CH), 0.5% iodine potassium iodide (IKI), and 0.5% cetrimide (CTR), alone and in combinations on one-week and three-week-old biofilms.

Materials and methods

Gingival plaque was collected, and biofilms were grown *in vitro* anaerobically. Biofilms were exposed to each of the three medicaments and their combinations for one day, one week, and two weeks. Proportions of dead and live bacteria in the biofilms were evaluated.

Results

The killing of bacteria by different medicaments in the three-week-old biofilm was lower than in the one-week-old biofilm (*p*<0.05). The efficacy of IKI and CTR in killing bacteria was weaker than that of CH, but the highest efficacy in killing was achieved when all three were combined (*p*<0.05). There was no significant difference in the antibiofilm effect between a day's exposure to the mixture of the three medicaments and one or two weeks of treatment with CH alone (*p*>0.05).

Conclusions

Three-week-old biofilms are more resistant to medicaments than one-week-old biofilms. Combining IKI and CTR with CH resulted in a stronger antibiofilm effect than using CH alone. Mixing the three medicaments may enable obtaining the desired clinical effect in a shorter exposure time.

## Introduction

Bacterial infections are the primary cause of apical periodontitis. The treatment of apical periodontitis primarily aims to control bacterial infections in the intricate root canal system, along with sealing the canal space to prevent reinfection [1]. Bacterial infections in the root canal are very prevalent in the form of sessile, dense communities referred to as biofilms, most of which should be removed by mechanical instrumentation and chemical irrigation, while the treatment of residual biofilms in the canal may rely on the use of interappointment intracanal medicaments [2-5].

The effectiveness of mechanical instrumentation and chemical irrigation is limited for a variety of reasons, including complex canal morphology (flattened canals, fins, anastomoses and isthmuses, lateral canals, etc.), which also prevents access to some sections of the root canal system [4-6]. Clinicians may choose to employ intracanal medication, typically from one to a few weeks, to further strengthen the antimicrobial effect of the treatment. This is particularly common in cases with acute symptoms and the presence of pus and inflammatory exudate in the canals [7,8]. Also, finishing the root canal treatment in a single visit is not always feasible due to time constraints, and medication is then injected into the root canal space.

Unlike intracanal interappointment medicine, irrigants have received significant attention in recently published studies. For many years, calcium hydroxide (CH) paste has been the choice of intracanal medication, either in pure form or together with a vehicle and radiopaque compound, providing alternative options for treatment. CH has demonstrated its effectiveness in eradicating a wide range of microorganisms when applied as a seven-day dressing [9]. Nevertheless, it is important to note that *Enterococcus faecalis (E. faecalis)*, the bacterium most frequently found in failed endodontic cases [10,11], has displayed a remarkable ability to persist within dentinal tubules even after prolonged exposure to CH treatment [12,13].

Studies on the antibacterial efficacy of iodine compounds have indicated that the use of iodine alone is ineffective; however, the addition of potassium iodide to iodine (IKI) has demonstrated effective antibacterial activity [14-17]. One limitation of IKI is its inability to dissolve tissue, and some patients may experience allergic reactions to specific iodine compounds. Nevertheless, tissues have exhibited excellent tolerance to IKI [14-16]. IKI has exhibited the capacity to fully eliminate *E. faecalis *from bovine root dentin when applied with a 15-minute contact time [14]. Therefore, the combined use of CH and IKI is expected to yield a significantly enhanced antibacterial efficacy.

Cetrimide (CTR), a cationic surfactant possessing bactericidal properties, has been shown to reduce the surface tension of solutions and exhibit a prolonged residual effect [17-22]. The combined use of CH and CTR is expected to result in a prolonged antibacterial effect. An *in vitro* study evaluating CTR's effectiveness in combination with various chelating agents has revealed a reduction in the regrowth of *E. faecalis *for up to 60 days. However, it has been suggested that CTR's peak activity occurs between 15 and 20 days [22]. Previous studies have suggested enhanced bactericidal effect with the combination products in the main root canal (*in vivo*) or infected dentin (*in vitro*) using bacterial culturing and colony counting method [23-25], while other studies have not detected a difference in effectiveness [26,27]. To the best of our knowledge, no studies about the effect of CH mixed with different medicaments against biofilms using viability staining and confocal laser scanning microscope (CLSM) imaging have been conducted so far. This study aimed to investigate the time-dependent antibiofilm effect on one- and three-week-old oral biofilms by CH, CTR, and IKI used in isolation and various combinations.

## Materials and methods

Biofilm model

The University of British Columbia Clinical Research Ethics Board (certificate no: H12-02430) approved the protocol of this study. As described in our previous studies [23-26], sterile hydroxyapatite (HA) disks were used as substrate to grow biofilms. The HA disks were coated overnight at 4 °C with bovine dermal type I collagen (10μg/mL collagen in 0.012 N HCl in water; Cohesion, Palo Alto, CA) in each well of a 24-well plate. Written informed consent for collecting plaque bacteria was obtained. Supragingival plaque on the first or second upper molars was collected using sterile wooden tooth sticks from one adult volunteer with healthy gingiva and was suspended in brain-heart infusion (BHI) broth (Becton Dickinson, Sparks, MD). The bacterial suspension was adjusted to an optical density (OD) value of 0.08-0.10 (595 nm, 150 μL; polypropylene microtiter plates, Corning, NY). The prepared collagen-coated HA (C-HA) disks were rinsed in potassium-buffered solution (PBS) and then immersed in 1.8-mL BHI broth and 0.2 mL of the dispersed dental plaque solution in a 24-well plate. The plate was incubated under anaerobic conditions (AnaeroGen; Oxoid, Hampshire, UK) at 37 °C for one and three weeks. Fresh BHI broth was changed once every week for the aged biofilm.

Medicaments

All medicaments used in the experiment were prepared freshly at standard concentrations (Table 1).

**Table 1:** Medicament concentrations and mixing ratios CH: calcium hydroxide; CTR: cetrimide; IKI: iodine potassium iodide

Medicaments	Ingredients	Ratios
1% IKI	4% IKI + H_2_O	1:3
0.5% IKI	1% IKI + H_2_O	1:1
0.5% CTR	1% CTR + H_2_O	1:1
0.5% (IKI + CTR)	1% IKI + 1% CTR	1:1
CHpaste	CH powder + H_2_O	1:2
CHpaste + 0.25% IKI	CH powder + 0.5% IKI	1:1
CH paste + 0.25% CTR	CH powder + 0.5% CTR	1:1
CH paste + 0.25% (IKI + CTR)	CH powder + 0.5% (IKI + CTR)	1:1

This ensured consistent and reliable results throughout the experiment. CTR (Sigma-Aldrich, St. Louis, MO) and IKI (Carolina Biological Supply Company, Burlington, NC) were made to a final concentration of 0.5% (w/v) by diluting 4% IKI and 1% CTR stock solutions in distilled water [17]. This step ensures that the final concentration of the medications is standardized and can be reproduced throughout the experiment. A thin paste was made by combining CH powder (Sigma-Aldrich) with distilled water in a 1:2 ratio (w/v); 0.5% CTR, 0.5% IKI, and 0.5% (CTR + IKI) were set to a final concentration of 0.25% when mixed with CH powder. 

Exposure to medicaments

Biofilms at different stages of maturation (one week and three weeks) were utilized to evaluate the killing of biofilm bacteria by the individual medications and their mixtures. Each group was allocated two discs with five sampling sites each (a total of 10 experiments per group). As a positive control, untreated biofilms on C-HA discs were employed, and each medication was evaluated on both one-week and three-week-old biofilms for various treatment times. The biofilms were exposed to the medications and their mixtures for 24 hours, one week, and two weeks. In a 24-well tissue culture plate, the C-HA discs were submerged in 1 mL of the prepared medications. All experiments were carried out under anaerobic conditions at 37 °C.

Confocal laser scanning microscope analysis

Before viability staining and CLSM imaging, the biofilms were initially subjected to the medicaments for the given times before being washed with 0.85% NaCl solution for one minute to remove the medication. The discs were then stained by using the LIVE/DEAD Backlight Bacterial Viability kit (Molecular Probes, Eugene, OR) [28-31] as per the manufacturer’s instructions. To prevent any bias in the selection of images that were red or green, five randomly chosen locations on each disc to be scanned were chosen before the fluorescence was turned on. Green (living) and red (dead) fluorescence from labeled cells were seen utilizing simultaneous dual-channel imaging and CLSM (Nikon Eclipse C1; Nikon Canada, Mississauga, Canada). The mounted specimens were examined using a 10× lens, with a field of view of 1.64 mm^2^. Five randomly selected areas were scanned per disc, yielding a total of 10 scanned areas for each group. Imaris 7.2 (Bitplane Inc., St. Paul, MN) software was used to analyze the CLSM image stacks.

Data analysis

The mean volume of live and dead bacteria cells was calculated. The number of image stacks was also used to determine the biofilm thickness. The Imaris software was used to recreate a 3D biofilm structure. The antibacterial impact of each medicament was measured by the proportion of the volume of dead cells in each group. Since the data were not normally distributed, the significance of the results was tested using non-parametric tests (SPSS Statistics V.21; IBM Corp., Armonk, NY). The Kruskal-Wallis test was used with dependent variables between groups. Mann-Whitney U test was used to compare independent variables (across various medicament groups and between one-week and three-week-old biofilms).

## Results

**Figure 1:**
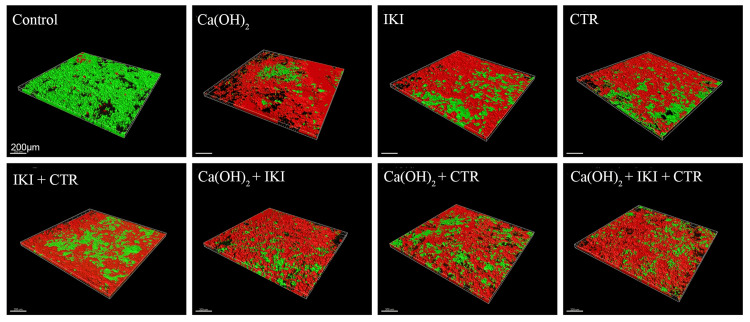
3D Reconstruction of CLSM images of one-week-old biofilms exposed to various medicaments for two weeks The medicament(s) used, and the percentage of dead bacteria cell volume are shown. The red color shows killed bacteria cells; the green color shows live bacteria cells CLSM: confocal laser scanning microscope; CTR: cetrimide; IKI: iodine potassium iodide

**Figure 2:**
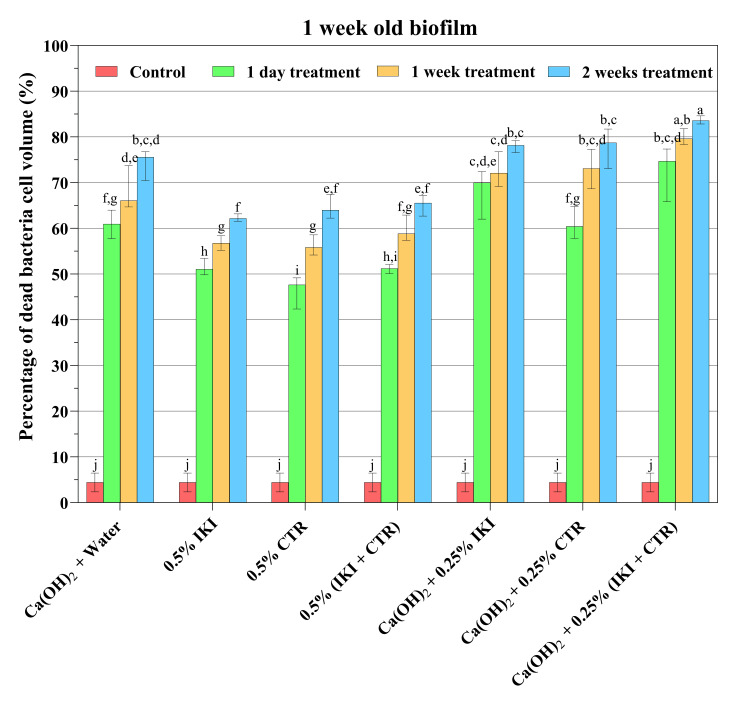
Percentage of dead bacteria cell volume (median, maximum, and minimum values) in one-week-old biofilms after the indicated treatments Different lowercase letters indicate statistically significant differences among each dataset across different medications and experimental periods (*p*<0.05) CTR: cetrimide; IKI: iodine potassium iodide

**Figure 3:**
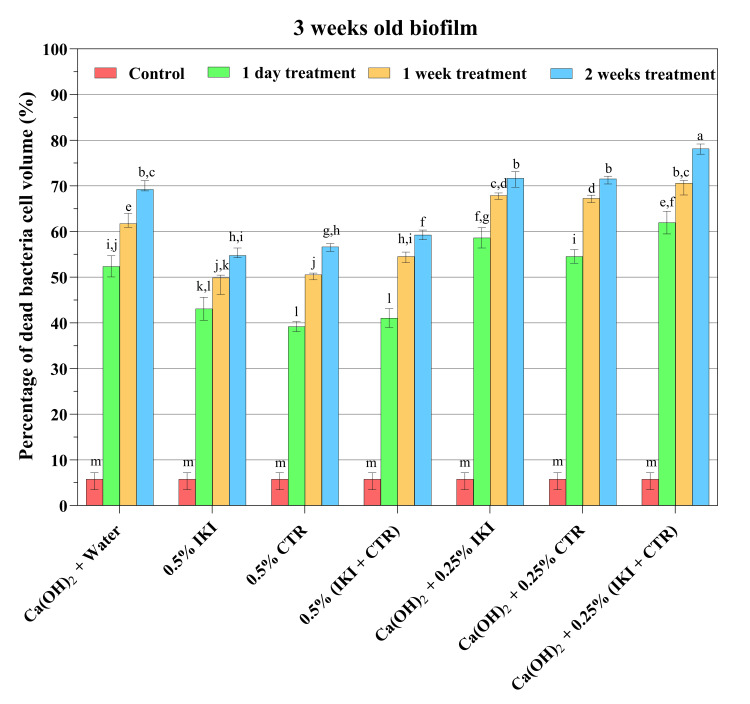
Percentage of dead bacteria cell volume (median, maximum, and minimum values) in three-week-old biofilms after the indicated treatments Different lowercase letters indicate statistically significant differences among each dataset across different medications and experimental periods (*p*<0.05) CTR: cetrimide; IKI: iodine potassium iodide

Figures 1-3 present a summary of the proportion of dead and live bacteria mass in CLSM and the percentage of dead bacteria cell volume (median, maximum, and minimum values) in one- and three-week-old biofilms for each group. After just one day of medication exposure, all medication treatments considerably outperformed the positive control in terms of the proportion of dead bacteria cells (*p*<0.05). The dead bacteria cell volume of the one- and three-week-old biofilms in the positive control groups did not differ statistically (*p*>0.05). With increased medication exposure duration, the proportion of dead bacteria cells increased across all treatment groups in a statistically significant manner (*p*<0.05; Figures 2-3). The killing of bacteria cells by different medicaments in the three-week-old biofilm was lower than in the one-week-old biofilm (*p*<0.05; Figures 2, 3).

After one day of exposure, the killing percentage ranged from 38.46% to 79.15% (median: 59.61%) in one-week-old biofilms, and from 37.89% to 66.49% (median: 52.08%) in three-week-old biofilms, depending on the medicaments used (Figures 2-3). After one week of exposure, the corresponding numbers were 53.74% to 84.06% (median: 65.73%) and 45.50% to 72.47% (median: 61.75%), and after two weeks of exposure, they were 60.68% to 85.48% (median: 65.73%) and 53.53% to 79.66% (median: 69.09%). At each time point, one day and one and two weeks, the largest proportion of dead bacteria cells of any treatment was observed with the mixture of CH, IKI, and CTR (*p*<0.05). There was no significant difference in the antibiofilm effect between one day's exposure to the mixture of the three medicaments and one or two weeks of treatment with CH alone (*p*>0.05).

## Discussion

To the best of our knowledge, this is the first study to use viability staining and CLSM imaging to assess the impact of CH when used alone or in combination with two other antibacterial agents on oral multispecies biofilms at different stages of maturation. The benefit of the model used is that the biofilm can be "cloned" and multiplied from the same source biofilm to produce a virtually endless number of parallel biofilm samples grown under the same conditions [28-32]. This is likely to improve the reliability of the results and comparisons between different medicaments. The finding supports that of a prior study utilizing the same model with different medications, which found that older biofilms are more resistant to antimicrobial medications compared to younger biofilms [33]. In positive controls, the proportion of dead bacteria cell volume in young and old biofilms was not different. The higher resistance of the older biofilms to medicaments could be due to the maturation of the biofilm extracellular polymeric substance and lower metabolic activity of the microbial cells [29,33]. The three-week-old biofilms were only slightly thicker than the one-week-old biofilms, with no statistically significant difference.

CH has for decades been the de facto standard root canal medicament between appointments in two- and multi-appointment root canal treatments [7,8]. The results of the present study showed a strong effect of CH on the biofilms; one-day exposure killed 52.08% and 60.95% of the bacteria cells in three-week and one-week-old biofilms, respectively. The proportion of killed bacteria rose additionally ca. 14.63% and 11.15% during the first and second weeks of continued exposure. These results, despite relatively high killing rates by CH, do not support the early clinical studies of the CH effect, which claimed the complete killing of root canal microbes [34]. However, the present results lend support to newer *in vivo* studies, which indicated that it was not possible to kill all bacteria with intracanal CH medication [35,36].

Results from the *in vivo* studies can be explained in several different ways, including the challenge of getting CH into every part of the root canal system. The present *in vitro* study showed that even in direct contact with the biofilm, CH, like other medicaments in this and other recent studies using the same model, was not able to kill all bacteria in the biofilm. The result emphasizes the importance of using biofilm models, mimicking the i*n vivo* situation, rather than planktonic cultures when the effect of disinfecting agents is examined [28]. Jardine et al. employed an intriguing *in situ* bacterial biofilm model to assess the antimicrobial efficacy of different bioceramic cements. They chose to use multispecies biofilms on infected human dentin blocks to replicate the genuine complexity and diversity of endodontic biofilms [37]. Furthermore, the result strongly supports emphasizing combined antimicrobial strategies of root canal treatment: instrumentation, irrigation, disinfection, and sealing of the canal space by a high-quality root filling using an antimicrobial sealer, and coronal restoration [38-41].

The efficacy of killing by CTR and IKI was slightly lower than that by CH at each corresponding time point, yet it achieved a substantial efficacy of killing against both old and young biofilms after two weeks of exposure. The effect of the combination of CTR and IKI did not differ from when they were used alone. CTR is typically used in low concentrations of 0.01-0.1%; in the present study, the concentration was 0.5% [17-20]. IKI, on the other hand, is usually used in a higher concentration of 2% (iodine) in 4% (potassium iodide) in the clinic. The reason for the lower concentration of 0.5% in the present study and previous studies with similar methodology was that higher iodine concentrations mask the fluorescence in CLSM [14-16,32]. Therefore, the killing efficacy of IKI in this study may be lower than what can be obtained with a 2/4% solution.

Previous studies have established the antimicrobial efficacy of CH mixed with various endodontic irrigating solutions or chelating agents [17-21]. Turk et al. conducted a study to explore the antimicrobial activity of CH when mixed with different vehicles (such as glycerin, chlorhexidine gluconate, CTR, and distilled water) against *E. faecalis* and *Candida albicans*, using the agar diffusion method [42]. The results of this study demonstrated that the combination of 2% CTR with CH powder produced a more potent antimicrobial effect against *E. faecalis* compared to using pure CH paste. In the present study, although the antibacterial impact of CH on biofilms did not increase by adding CTR after exposure for one day when compared to CH alone, the improved effect was statistically significant after one and two weeks of exposure. The higher efficacy of killing may have been caused by the CTR's residual effect, as shown in a prior study [22].

*E. faecalis* is commonly associated with persistent endodontic infections [4]. Sirén et al. have revealed that the use of CH alone was not effective in eliminating *E. faecalis *within bovine dentin blocks [23]. On the other hand, when CH was combined with IKI, effective disinfection of the dentin was achieved. Importantly, the addition of IPI does not impact the alkalinity of the CH suspensions, maintaining its antimicrobial properties intact. These findings suggest that the combination of CH and IPI can be a promising approach for disinfection in endodontic treatment, specifically against *E. faecalis* infections. In the present study, the antibiofilm impact was enhanced when IPI was added to CH or when all three agents were utilized after exposure for one day, one week, or two weeks. The strongest effect against the biofilm was produced by the mixture of CH, CTR, and IKI; depending on the age of the biofilm, it was as effective against the biofilm bacteria after a short term as pure CH was after long-term treatment (Figures 2, 3). Given that the same antibiofilm impact may be attained in such a brief period, this could represent a substantial advantage in practical practice.

This study has certain limitations. Firstly, only one source was utilized to create the multispecies biofilm. This decision was made to manage the complexity arising from the numerous variables involved, such as the various combinations of three antibacterial substances, three different exposure durations (one day, one week, and two weeks), and biofilms at two different maturation stages. Nevertheless, it is important to acknowledge that the absence of biofilm sources from multiple donors could be considered a potential limitation of the study. However, Stojicic et al. [33] have demonstrated that biofilms originating from gingival plaque samples obtained from six different sources (donors) exhibited surprisingly similar susceptibility behavior when exposed to three distinct disinfecting agents: chlorhexidine, sodium hypochlorite, and IKI. This finding suggests that there may be certain universal characteristics inherent to biofilms that play a more significant role in determining their susceptibility and resistance to disinfecting agents commonly used in endodontics, as opposed to the specific composition of individual biofilm species.

In another study, Zhang et al. assessed the impact of DJK-5, a D-Enantiomeric Peptide, on oral multispecies biofilms originating from three different donors [43]. The experiment involved cultivating these biofilms on HA discs, which were developed using plaque samples taken from each of the three donors. Interestingly, the study found that the susceptibility of these multispecies biofilms to DJK-5 alone, as well as to a combination of DJK-5 and 2% chlorhexidine, was consistent across all samples, irrespective of their origin. This consistency in response suggests that the differences in the source of the biofilms and the potential variations in their species composition did not significantly influence their reaction to the treatment. No published studies have shown different susceptibility to the same medications by *in vitro* biofilms grown from plaque from different donors. Nevertheless, the results of the present study must be interpreted with caution, although it is plausible that these findings could extend beyond the specific biofilm examined and potentially reflect a broader reality regarding the response patterns of biofilms to disinfection measures. 

Secondly, CLSM was employed to quantitatively assess the bacterial killing efficiency of CH, 0.5% IKI, and 0.5% CTR, both individually and in combination. While CLSM is a sophisticated imaging tool that facilitates precise, non-invasive, and in-depth evaluation of bacterial biofilms in three dimensions and at high resolutions, it does not provide detailed information on the surface morphology of bacterial biofilms. Understanding the surface morphology is crucial for a comprehensive analysis of biofilm integrity and bacterial cell morphology after exposure to medications. Thirdly, the C-HA disc biofilm model used in this study provided a standardized and effective method for comparing the bacterial killing efficiency of various medications. However, for a more clinically relevant assessment, models such as the dentin block model are recommended to better evaluate the application of different medicaments in a specific clinical context.

## Conclusions

Based on our findings, three-week-old biofilms are more resistant to medicaments than one-week-old biofilms. CH, IKI, and CTR when used as a mixture proved to have an additive antibiofilm action. One day of exposure to the mixture of the three medicaments had an antibiofilm effect comparable to one or two weeks of treatment with CH alone.
